# The rural Uganda non-communicable disease (RUNCD) study: prevalence and risk factors of self-reported NCDs from a cross sectional survey

**DOI:** 10.1186/s12889-021-12123-7

**Published:** 2021-11-07

**Authors:** Trishul Siddharthan, Robert Kalyesubula, Brooks Morgan, Theresa Ermer, Tracy L. Rabin, Alex Kayongo, Richard Munana, Nora Anton, Katharina Kast, Elke Schaeffner, Bruce Kirenga, Felix Knauf, Rural Uganda Non Communicable Disease Study Investigators, Trishul Siddharthan, Robert Kalyesubula, Asghar Rastegar, Theresa Ermer, Tracy L. Rabin, Alex Kayongo, Faith Nassali, Richard Munana, Nora Anton, Katharina Kast, Elke Schaeffner, Bruce Kirenga, Helmut Kraus, Felix Knauf

**Affiliations:** 1 Division of Pulmonary and Critical Care, School of Medicine, University of Miami, 1951 NW 7th Ave, Suite 2308, Miami, FL 33136 USA; 2 Division of Pulmonary and Critical Care, School of Medicine, Johns Hopkins University, Baltimore, MD USA; 3 Makerere College of Health Sciences, Makerere University, Kampala, Uganda; 4 African Community Center for Social Sustainability (ACCESS), Nakaseke, Uganda; 5 Faculty of Medicine, Friedrich-Alexander-Universität Erlangen-Nürnberg, Erlangen, Germany; 6 Department of Internal Medicine, Yale University School of Medicine, New Haven, CT USA; 7 Uganda Initiative for Integrated Management of Non-Communicable Diseases, Kampala, Uganda; 8 World Health Summit c/o Charité Universitätsmedizin Berlin, Berlin, Germany; 9 Department of Nephrology and Medical Intensive Care, Charité Universitätsmedizin Berlin, Berlin, Germany; 10 Institute of Public Health, Charité Universitätsmedizin Berlin, Berlin, Germany

**Keywords:** Non-communicable diseases, Rural, Low- and middle-income countries

## Abstract

**Background:**

Non-communicable diseases (NCDs) are an increasing global concern, with morbidity and mortality largely occurring in low- and middle-income settings. We established the prospective Rural Uganda Non-Communicable Disease (RUNCD) cohort to longitudinally characterize the NCD prevalence, progression, and complications in rural Africa.

**Methods:**

We conducted a population-based census for NCD research. We systematically enrolled adults in each household among three sub-counties of the larger Nakaseke Health district and collected baseline demographic, health status, and self-reported chronic disease information. We present our data on self-reported chronic disease, as stratified by age, sex, educational attainment, and sub-county.

**Results:**

A total of 16,694 adults were surveyed with 10,563 (63%) respondents enrolled in the self-reported study. Average age was 37.8 years (SD = 16.5) and 45% (7481) were male. Among self-reported diseases, hypertension (HTN) was most prevalent (6.3%). 1.1% of participants reported a diagnosis of diabetes, 1.1% asthma, 0.7% COPD, and 0.4% kidney disease. 2.4% of the population described more than one NCD. Self-reported HTN was significantly higher in the peri-urban subcounty than in the other two rural sub-counties (*p* < 0.001); diagnoses for all other diseases did not differ significantly between sub-counties. Odds for self-reported HTN increased significantly with age (OR = 1.87 per 10 years of age, 95% CI 1.78–1.96). Male sex was associated with lower odds of reporting asthma (OR = 0.53, 95% CI 0.34–0.82) or HTN (OR = 0.31, 95% CI 0.26–0.40).

**Conclusions:**

The RUNCD will establish one of the largest NCD patient cohorts in rural Africa. First analysis highlights the feasibility of systematically enrolling large numbers of adults living in a rural Ugandan district. In addition, our study demonstrates low levels of self-reported NCDs compared to the nation-wide established levels, emphasizing the need to better educate, characterize, and care for the majority of rural communities.

**Supplementary Information:**

The online version contains supplementary material available at 10.1186/s12889-021-12123-7.

## Background

The global prevalence of non-communicable diseases (NCDs) has risen over the past decade, especially in low- and middle-income countries (LMICs). As the global population ages, the total number of people affected by NCDs in LMICs will increase even further. The most prevalent NCDs in these settings include cardiovascular diseases, cancers, chronic respiratory diseases, type 2 diabetes mellitus (DM), and chronic kidney disease (CKD) [1, 2]. Many of these NCDs share a set of modifiable risk factors, such as tobacco use, obesity, poor diet, physical inactivity, and alcohol abuse [1]. Alongside rapid globalization and urbanization across LMIC settings these risk factors have become more prevalent, resulting in a dual burden of infectious and non-communicable diseases [1]. Furthermore, the current COVID-19 pandemic has illustrated that concomitant NCDs are the major risk factor for severe infectious-disease morbidity and mortality [3]. Therefore, it is not surprising that 85% of premature deaths related to NCDs occur in LMICs [4].

Although global burden of disease estimates exist for the prevalence of NCDs in LMIC settings, a number of these estimates are based on single-site studies [5]. In Uganda, a range of estimates regarding the NCD prevalence exists solely from/based on population-based surveys. A recent survey utilizing the WHO STEPS tool across Uganda demonstrated a 26.4% prevalence for hypertension (HTN), particularly in central Uganda at 28.5% [6]. A similar survey concerning DM demonstrated a prevalence of 1.4% [7]. Although no national estimates exist for chronic respiratory disease, previous estimates for chronic obstructive pulmonary disease (COPD) have ranged from 1.5% in urban areas to 16.2% in a rural setting [8, 9]. Asthma prevalence estimates range from 9.7% in urban settings to 4.4% in rural settings [10]. A population-based study in Uganda demonstrated high levels of early-stage kidney disease, which does not appear to be driven by the traditional risk factors [11].

Multimorbidity or NCD management strategies require prospective, longitudinal data. For instance, prospective cohort studies on communicable diseases in Uganda have substantially advanced our knowledge about HIV-AIDS and have made major contributions to improve the care and management of affected patients [12, 13]. Following this model, we propose to establish The Rural Uganda Non-Communicable Disease study (RUNCD) as a longitudinal cohort of individuals with NCDs. Our long-term goals are to improve our understanding of NCDs, identify risk factors for progression, develop predictive models to classify high-risk subgroups, and build a platform for collaborating researchers to jointly conduct preventive therapies and future treatment trials. In addition, we believe that such a study can serve as a major educational tool for the national and international academic community to improve the care for patients with NCD. We present the RUNCD baseline data concerning population demographics, self-reported disease, and risk factors.

## Methods

### Study design, setting, and data collection

**Fig. 1:**
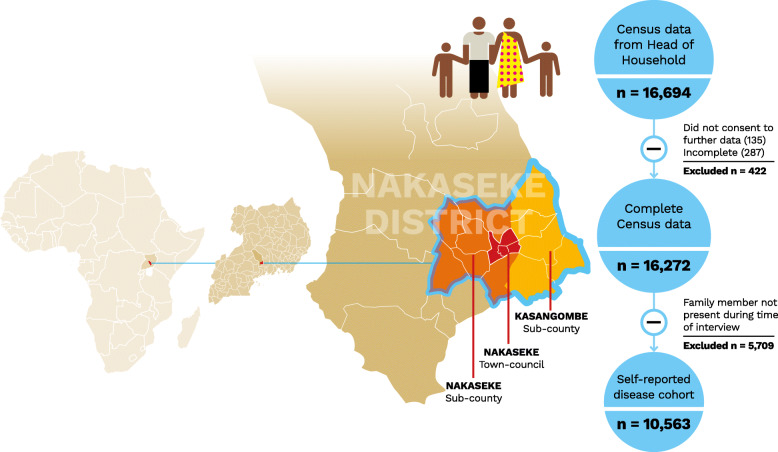
Selected sub-districts of Nakaseke. The highlighted areas represent Nakaseke Sub-county (left), Nakaseke Town Council (center), and Kasangombe Sub-county (right). The flow chart demonstrates the number of respondents included in the study. (Designed by Helmut Kraus, permission obtained)

A population census in three sub-counties of the larger Nakaseke Health District was performed as a baseline to develop a prospective observational RUNCD study site to characterize the manifestation, progression, and complications of NCDs in rural Africa. The three sub-counties of Kasangombe, Nakaseke, and Nakaseke Town Council were purposively selected for the study based on their differing degrees of urbanization and age distribution (Fig. 1). Nakaseke Town Council had the highest rate of urbanization with 309 persons/km^2^, followed by Kasangombe (194 persons/km^2^), and Nakaseke (175 persons/km^2^) sub-counties [14].

Between 2016 and 2017, trained fieldworkers and community health workers (CHWs) went door-to-door and administered a household census among all adults above age 18 years in each of the three sub-counties. Heads of household were surveyed to provide sociodemographic information on behalf of all adults living in the household, and all adults present in the house at the time of sampling received self-reported chronic disease questionnaires for HTN, DM, asthma, COPD, and CKD (Supplement).

### Stakeholder engagement

Local Nakaseke district authorities as well as the Mulago Research Ethics Committee and the Uganda National Council for Health, Science, and Technology approved the study protocol (SS 4283). Simplified versions of the study protocol were discussed and shared with the local council chairpersons, the District Health Officer, and the Resident District Commissioner. Other stakeholders from lower level health units were also informed about the study through a series of community engagement meetings. A community advisory committee was set up to ensure continuous involvement in the project and assist with the generation of locally relevant research questions on NCDs. CHWs, assigned to villages within the study area, were trained in research and electronic data capture.

### CHW training and baseline survey

We held a three-day training for the CHWs aimed at equipping CHWs with basic knowledge on NCDs and research skills. The training followed our two-day modular curriculum developed in conjunction with the Ugandan Ministry of Health. The training equipped the CHWs with skills in detection and referral of NCDs and health promotion. CHWs accompanied field workers to households to assess self-reported estimates of HTN, DM, asthma, COPD, and kidney disease. Each was defined by a positive response to the question: “Have you ever been diagnosed with any of the following health conditions?” Standard questions developed for each of the chronic diseases under study were administered according to the disease of interest [9].

### Data management

In partnership with the Uganda Bureau of National Statistics, enumerated areas including villages and individual dwellings were mapped and individuals living within households were assigned unique anonymous identification numbers. These identifiers will be retained throughout the study and will be linked throughout the different studies to other collected data, including laboratory tests. Data were captured at the time of the interview utilizing tablet computers programmed with Research Electronic Data Capture (REDCap; Vanderbilt University, Nashville, USA).

### Biostatistical methods

Demographic factors were compared between sub-counties by ANOVA for continuous variables or chi-squared tests for dichotomous variables. For each chronic disease, a prevalence and 95% confidence interval (CI) were generated. Risk factors for chronic disease in this region were analyzed by performing multivariable logistic regression. The chronic disease of interest was modeled as the outcome, while age, sex, educational attainment, and sub-county were included as risk factors. Next, we tested interactions by age and sex to see if the risks differed between males and females of varying ages. All analyses were performed in STATA version 13 (StataCorp, College Station, USA) and R (www.r-project.org).

## Results

### Baseline characteristics

**Fig. 2:**
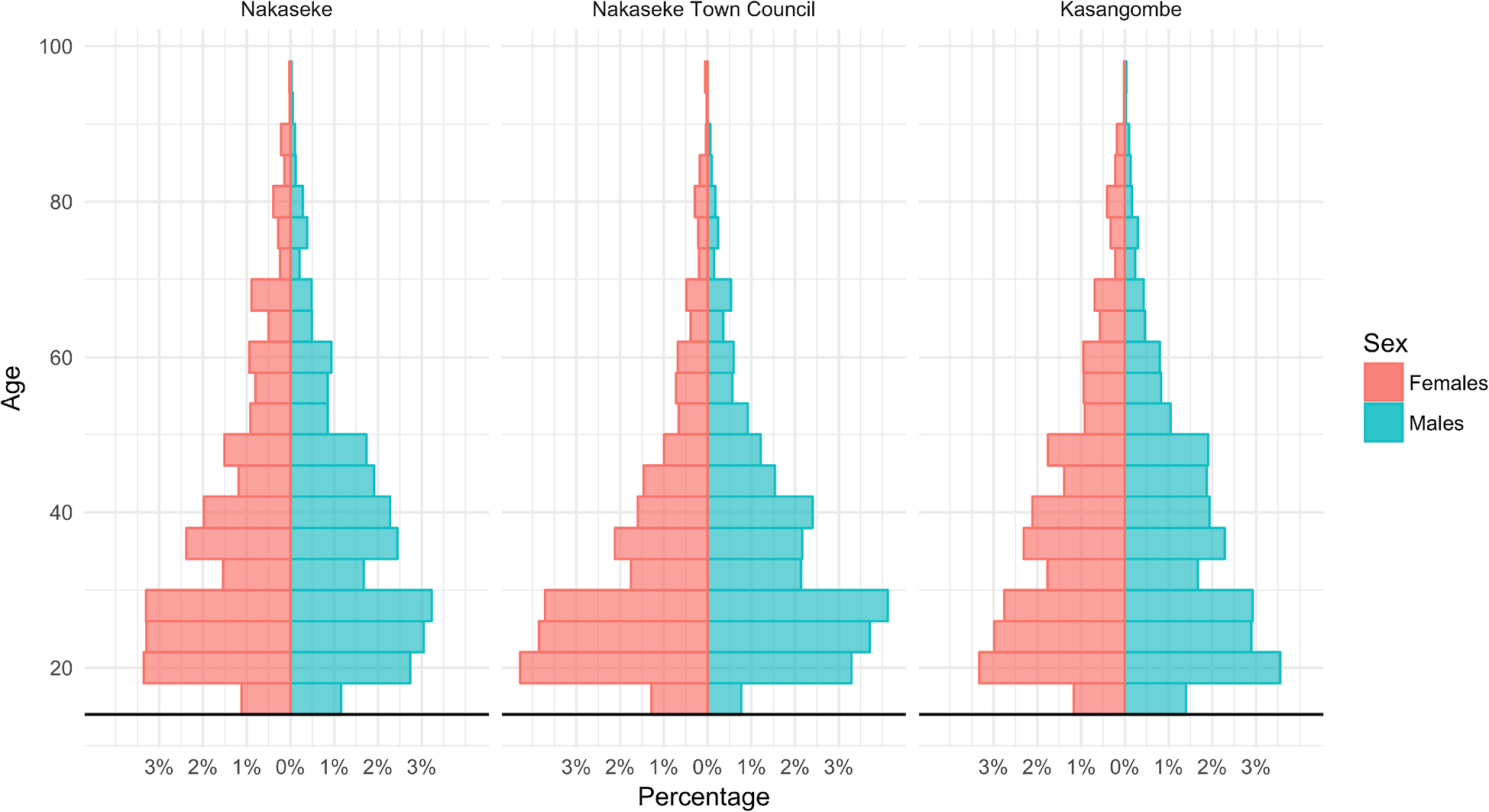
Demographics of sub-counties in Nakaseke. The distribution of female respondents and male respondents in Nakaseke, Nakaseke Town Council, and Kasangombe stratified by age groups

The census captured data on 16,694 participants from Kasangombe, Nakaseke sub-county, and Nakaseke Town Council. A total of 135 participants declined to consent and the overall response rate of the census among heads of household was 99.2%. A further 287 were excluded due to incomplete basic demographic data, resulting in 16,272 participants included in the final census. For the analysis of self-reported chronic disease prevalence, 5709 were not included due to not being present at the time of the interview, leaving a sample of 10,563 as summarized in Fig. 1. Of those included in the initial analysis, 43.1% (*n* = 7006) resided in Nakaseke sub-county, 13.1% (*n* = 2128) resided in Nakaseke Town Council, and 43.9% (*n* = 7138) resided in Kasangombe. Overall, 45.3% were male and the average age of participants was 37.8 years (Table 1, Fig. 2). The majority of participants had completed primary or less education (66.0%. *n* = 10,995) and reported being employed at the time of the interview (70.1%, *n* = 11,376). Total household size was 5.1 (SD = 3) people on average.

**Table 1:** Sociodemographic characteristics of the census from the sampled sub-counties

	Nakaseke	Nakaseke Town Council	Kasangombe	Total	***p***-value
Percent of Population (n)	43.1% (7006)	13.1% (2128)	43.9% (7138)	16,272	
Mean Age (SD)	38.3 (16.7)	35.3 (15.3)	38.1 (16.6)	37.8 (16.5)	< 0.001
Male Sex (n)	45.1% (3206)	39.5% (855)	47.1% (3420)	45.3% (7481)	< 0.001
Mean Household Size (SD)	5.0 (3.0)	4.7 (3.2)	5.3 (3.0)	5.1 (3.0)	< 0.001
**Education**					
Primary or Less (n)	71.3% (5098)	50.5% (1105)	65.6% (4792)	66.0% (10,995)	< 0.001
**Employment Status**					
Currently Employed (n)	66.5% (4643)	75.8% (1604)	71.9% (5129)	70.1% (11,376)	< 0.001

Of the 10,563 included in the analysis of chronic disease, HTN was the most frequently reported 6.3% (*n* = 659) (Tables 2 & 3, Supplement Fig. 1). However, the self-reported prevalence of HTN differed significantly between the sub-counties (*p* < 0.001), being highest in Nakaseke Town Council at 8.5% (*n* = 118), followed by Kasangombe at 6.8% (*n* = 298), and Nakaseke sub-county at 5.2% (*n* = 243). About 1.1% (*n* = 116) of participants reported diagnosis of DM, 1.1% (*n* = 116) asthma, 0.7% (*n* = 77) COPD, and 0.4% (*n* = 41) kidney disease. About 2.4% (*n* = 256) of the population reported more than one NCD. The self-reported prevalence of DM, asthma, COPD, and kidney disease was not significantly different among the three sub-counties.

**Table 2:** Prevalence of self-reported disease by sub-county

	Nakaseke	Nakaseke Town Council	Kasangombe	Total	***p***-value
Percent of Respondents (n)	44.7% (4722)	13.3% (1401)	42.0 (4440)	10,563	
**Disease Prevalence: % (n)**					
Hypertension	5.2% (243)	8.5% (118)	6.8% (298)	6.3% (659)	< 0.001
Diabetes Mellitus	1.0% (45)	1.2% (16)	1.3% (55)	1.1% (116)	0.40
Asthma	0.9% (40)	1.1% (15)	1.4% (61)	1.1% (116)	0.05
COPD	0.6% (28)	0.5% (7)	1.0% (42)	0.7% (77)	0.08
Kidney Disease	0.3% (14)	0.4% (6)	0.5% (21)	0.4% (41)	0.37
Multimorbidity	1.5% (72)	4.3% (60)	2.8% (124)	2.4% (256)	< 0.001

**Table 3:** In-sample versus out-of-sample characteristics

	Included	Excluded	***p***-value
Percent of Population (n)	63.3% (10,563)	36.7% (6131)	
Mean Age (SD)	39.2 (17.1)	35.1 (15.1)	< 0.001
Male Sex (n)	36.3% (3829)	61.1% (3670)	< 0.001
Mean Household Size (SD)	4.7 (2.9)	5.9 (3.1)	< 0.001
**Education**			
Primary or less (n)	70.3% (7392)	62.5% (3630)	< 0.001
**Employment Status**			
Currently Employed (n)	31.8% (3347)	27.1% (1646)	< 0.001

In Nakaseke Town Council, the 60- to 75-year-old age group had the highest self-reported chronic disease burden, particularly for DM, HTN, and multiple NCDs. In Nakaseke and Kasangombe sub-county the self-reported chronic disease burden was highest in the 75+ year-old age group (Table 2).

### Risk factors of self-reported NCDs

**Fig. 3:**
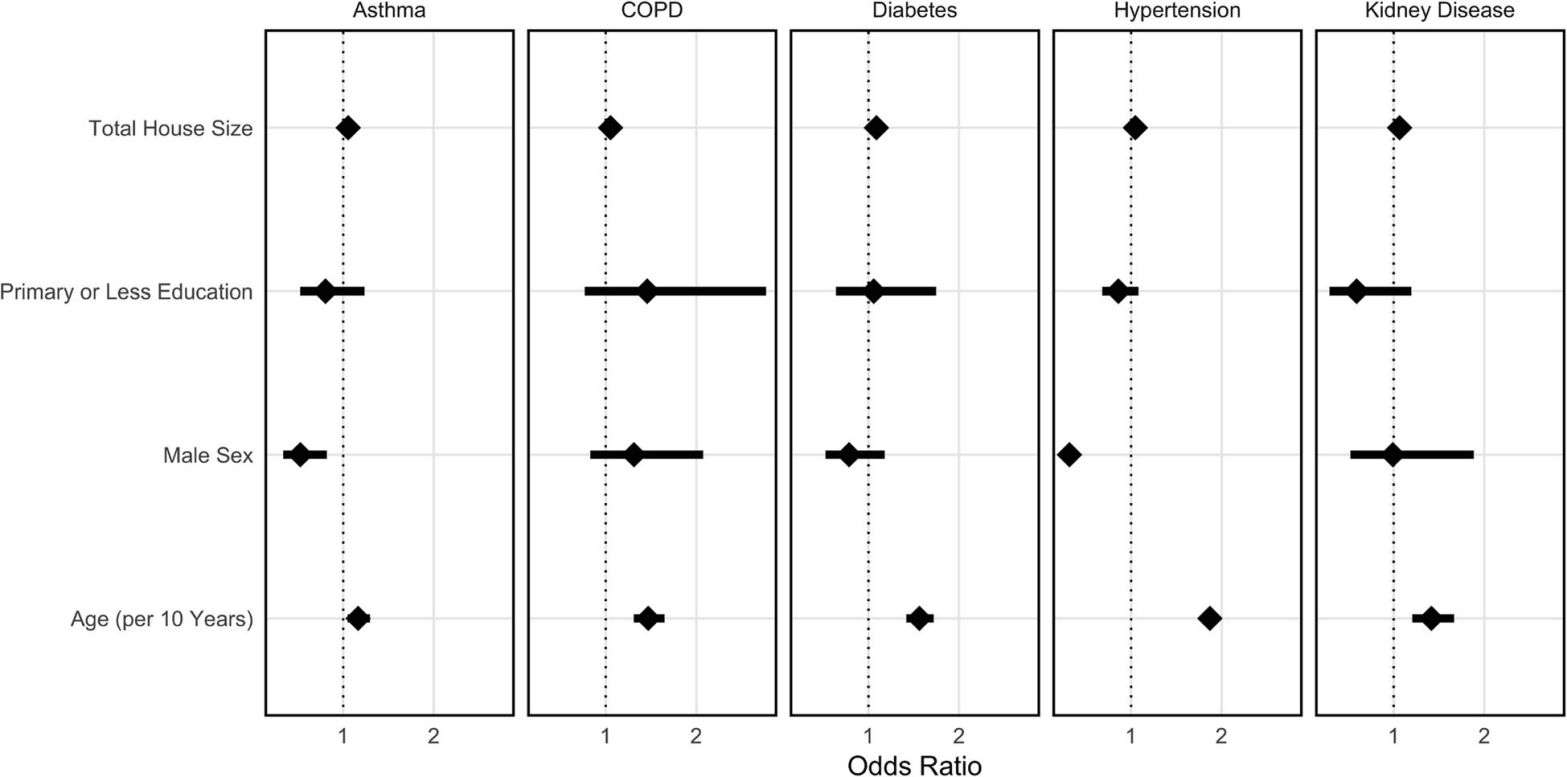
Association between risk factors (y-axis) and self-reported disease. Diamonds represent adjusted odds ratios with corresponding 95% CIs shown as error bars

We next determined odds ratios describing associations between total household size, education, sex, and age with HTN, DM, asthma, COPD, and kidney disease (Fig. 3). Odds ratios for self-reporting any of the diseases under consideration did not change with household size or education level but increased significantly with increasing age. Older age was most strongly associated with self-reported HTN (OR = 1.87 per 10 years of age, 95% CI 1.78–1.96). Male sex was associated with lower odds of reporting HTN (OR = 0.31, 95% CI 0.26–0.40) or asthma (OR = 0.53, 95% CI 0.34–0.82). Moreover, we examined the effect modification of gender on age. No interactions were found between age and sex for HTN, DM, COPD, or kidney disease. However, we did find a positive effect for increasing age and male sex among those with self-reported asthma (OR = 1.32, 95% CI 1.06–1.66). This indicates that the odds of self-reported asthma increase more quickly among men as age increases.

## Discussion

We present baseline census data for the RUNCD with the aim of developing a cohort of longitudinal studies to address gaps in knowledge, literacy, and care concerning NCDs in LMICs [1]. We found a high association between older age and self-reported HTN consistent with previous studies conducted in Uganda [6, 7, 15]. Self-reported prevalence of HTN was lower in men compared to women, which may reflect increased health seeking behavior and improved awareness among women rather than underlying gender differences in the prevalence of HTN. Self-reported HTN and DM was associated with older age similar to previous self-reported data, yet much lower as compared with national estimates [6, 7, 16]. We found the odds of self-reported asthma to increase more quickly among men as age increases but not with education level or household size. A range of risk factors for asthma has been described in LMIC settings, predominantly in urban areas. The trend we observed may, therefore, be the result of a combination of risk factors including increased ambient air pollution in urban environments, specific allergens, poor sanitation, and adoption of westernized lifestyle, all of which are associated with an increased inflammatory response [10]. The prevalence of self-reported kidney disease was very low (< 1%) in comparison to previously established Ugandan estimates [11]. Kidney disease has long been recognized as a silent epidemic [16, 17]. While several multinational kidney disease studies have been established in high income countries, none has included an African cohort [18]. As kidney disease requires formal laboratory testing for diagnosis, the low prevalence in our study likely represents an underdiagnosis of kidney disease in the setting of insufficient disease awareness and limited access to laboratory testing in these rural communities. This highlights the need to direct our capacity-building focus to these areas.

Our study has several strengths. First, we demonstrate the feasibility of using a CHW-approach to systematically capture residents in rural area of Uganda. CHWs in Uganda are part of the national health system and liaise between the Ministry of Health on national health programs, as well as local and regional hospitals to facilitate care [19]. While this position has previously been voluntary the Ugandan Ministry of Health has implemented a payment structure for CHWs.

The response rate of 99.2% is extremely high, which supports that CHWs may be ideally suited to be involved in research studies given the trust they receive in their community. Second, we assessed a range of self-reported NCDs in this population, with analysis of risk factors across diseases and multimorbidity. Third our sampling captured a younger overall population, similar to other census studies in rural Uganda [20]. Studies specific to rural areas are necessary to improve understanding of disease-burden and risk factors in these largely underserved areas.

Our study also has several potential shortcomings. First, we were only able to collect self-reported data on NCDs among 63% of respondents. Non-participation can skew the findings towards individuals who work and/or are unable to seek healthcare more readily thereby resulting in lower prevalence estimates. The described prevalence of self-reported disease is dependent on access to diagnostic equipment and health professionals, both of which are limited in rural Uganda. Diagnosis of NCDs is additionally limited by lack of records to verify diagnosis as well as recall bias among participants. Additionally, while overall lower, the prevalence of self-reported HTN was higher compared to DM, which could be a result of more accessible diagnostic equipment of knowledge of HTN. Second, self-reported disease is a proxy for health seeking behaviors, which are gender and age dependent [21]. Third, given the scope of this census, we were limited in the ability to fully assess risk factors for disease. Longitudinal studies assessing population-attributable risk require additional baseline assessments of traditional risk factors (e.g. tobacco, diet, alcohol). Further studies require detailed exposure history, and activity and diet as well as anthropometric measurements such as BMI, which have been shown to be associated with DM and HTN.

### Future directions

In resource limited settings, CHWs have been demonstrated to effectively increase the uptake of services for the prevention and management of NCDs and improve disease control through a patient-centered approach [1, 19, 22]. However, the basis for implementing an integrated approach to managing NCDs is an assessment of local disease prevalence through epidemiological research. We plan to use the RUNCD to create a platform for the characterization of NCDs in Sub-Saharan Africa to achieve four principal goals:

1. Characterize the prevalence of NCDs in a defined cohort of inhabitants who live in a rural area of Uganda using a CHW-approach. We will evaluate the accordance of self-reported disease status with confirmatory testing in order to elucidate factors associated with disease awareness and access to care while adjusting for confounders (i.e. age, sex, cigarette smoking, body mass index, secondary education).
2. Assess traditional and nontraditional risk factors associated with NCDs in rural Uganda. Nontraditional NCD risk factors are defined as factors that have not yet been well studied but may have a higher relevance in the Sub-Saharan setting, in contrast to well established factors such as tobacco, obesity and diet. We will utilize the WHO STEPS across a subset of participants to assess traditional risk factors.
3. Assess the implementation of comprehensive CHW-facilitated NCD management strategies in Nakaseke using CHWs currently incorporated within the Ugandan Ministry of Health’s (MOH) mission to deliver care in rural settings. Using a referral system, the patients will also be followed by physicians and nurses assigned to the designated villages.
4. Provide information on the diagnosis, knowledge, and effectiveness of NCD management in LICs that allows the formulation of hypotheses for targeted interventional trials that focus on reducing the burden and mortality related to NCDs.

## Conclusion

There is an urgent need to improve education and diagnostic capabilities among rural communities of Uganda and elsewhere for the prevention, early diagnosis, and effective management of NCDs. Therefore, we have established the RUNCD study that utilizes observational epidemiology to address research questions regarding the etiology, prognosis, health service utilization, and role of CHWs in improving the quality of life among patients with NCDs.

## Supplementary Information

**Additional file 1: Supplement Fig. 1.** Prevalence of self-reported non-communicable diseases in Nakaseke stratified by sex and age. The prevalence of self-reported asthma, COPD, diabetes, kidney disease, hypertension, or multimorbidity by 15-year increments stratified by sex. (Designed by Helmut Kraus, permission obtained)

## Data Availability

The datasets used and/or analyzed during the current study are available from the corresponding author upon reasonable request.

## Abbreviations

NCDs: Non-communicable diseases
LMICs: Low- and Middle-income Countries
DM: Diabetes mellitus
CKD: Chronic kidney disease
HTN: Hypertension
COPD: Chronic obstructive pulmonary disease
RUNCD: Rural Uganda Non-Communicable Disease study
HIV/AIDS: Human immunodeficiency virus infection and acquired immunodeficiency syndrome
CHW): Community health workers

## References

[1] Checkley W, Ghannem H, Irazola V, Kimaiyo S, Levitt NS, Miranda JJ, et al. Management of NCD in low-and middle-income countries. Glob Heart. 2014;9(4):431–43. 10.1016/j.gheart.2014.11.003. 10.1016/j.gheart.2014.11.003 PMC4299752 25592798

[2] Levin A, Tonelli M, Bonventre J, Coresh J, Donner JA, Fogo AB, et al. Global kidney health 2017 and beyond: a roadmap for closing gaps in care, research, and policy. Lancet. 2017;390(10105):1888–917. 10.1016/S0140-6736(17)30788-2. 10.1016/S0140-6736(17)30788-2 28434650

[3] Richardson S, Hirsch JS, Narasimhan M, Crawford JM, McGinn T, Davidson KW, et al. Presenting characteristics, comorbidities, and outcomes among 5700 patients hospitalized with COVID-19 in the new York City area. JAMA. 2020;323(20):2052–9. 10.1001/jama.2020.6775. 10.1001/jama.2020.6775 PMC7177629 32320003

[4] Alwan A (2011). Global status report on noncommunicable diseases 2010.

[5] Lopez AD, Murray CC (1998). The global burden of disease, 1990–2020. Nat Med.

[6] Guwatudde D, Mutungi G, Wesonga R, Kajjura R, Kasule H, Muwonge J, et al. The epidemiology of hypertension in Uganda: findings from the national non-communicable diseases risk factor survey. PLoS One. 2015;10(9):e0138991. 10.1371/journal.pone.0138991. 10.1371/journal.pone.0138991 PMC4583385 26406462

[7] Bahendeka S, Wesonga R, Mutungi G, Muwonge J, Neema S, Guwatudde D (2016). Prevalence and correlates of diabetes mellitus in Uganda: a population-based national survey. Tropical Med Int Health.

[8] van Gemert F, Kirenga B, Chavannes N, Kamya M, Luzige S, Musinguzi P, et al. Prevalence of chronic obstructive pulmonary disease and associated risk factors in Uganda (FRESH AIR Uganda): a prospective cross-sectional observational study. Lancet Glob Health. 2015;3(1):e44–51. 10.1016/S2214-109X(14)70337-7. 10.1016/S2214-109X(14)70337-7 25539969

[9] Siddharthan T, Grigsby MR, Goodman D, Chowdhury M, Rubinstein A, Irazola V, et al. Association between household air pollution exposure and chronic obstructive pulmonary disease outcomes in 13 low-and middle-income country settings. Am J Respir Crit Care Med. 2018;197(5):611–20. 10.1164/rccm.201709-1861OC. 10.1164/rccm.201709-1861OC PMC6005243 29323928

[10] Morgan BW, Siddharthan T, Grigsby MR, Pollard SL, Kalyesubula R, Wise RA, et al. Asthma and allergic disorders in Uganda: a population-based study across urban and rural settings. J Allergy Clin Immunol Pract. 2018;6(5):1580–7. 10.1016/j.jaip.2017.11.032. 10.1016/j.jaip.2017.11.032 PMC6050146 29361510

[11] Kalyesubula R, Nankabirwa JI, Ssinabulya I, Siddharthan T, Kayima J, Nakibuuka J, et al. Kidney disease in Uganda: a community based study. BMC Nephrol. 2017;18(1):1–9. 10.1186/s12882-017-0521-x. 10.1186/s12882-017-0521-x PMC5379733 28372551

[12] Sewankambo NK, Wawer MJ, Gray RH, Serwadda D, Li C, Stallings RY, et al. Demographic impact of HIV infection in rural Rakai district, Uganda: results of a population-based cohort study. AIDS. 1994;8(12):1707–13. 10.1097/00002030-199412000-00011. 10.1097/00002030-199412000-00011 7888120

[13] Asiki G, Murphy G, Nakiyingi-Miiro J, Seeley J, Nsubuga RN, Karabarinde A, et al. Sandhu MS; GPC team. The general population cohort in rural South-Western Uganda: a platform for communicable and non-communicable disease studies. Int J Epidemiol. 2013;42(1):129–41. 10.1093/ije/dys234. 10.1093/ije/dys234 PMC3600628 23364209

[14] Uganda Bureau of Statistics (2016). The National Population and Housing Census 2014 – Main Report, Kampala, Uganda.

[15] Balcázar H, Fernández-Gaxiola AC, Pérez-Lizaur AB, Peyron RA, Ayala C (2015). Improving heart healthy lifestyles among participants in a Salud para su Corazón promotores model: the Mexican pilot study, 2009-2012. Prev Chronic Dis.

[16] Stanifer JW, Muiru A, Jafar TH, Patel UD (2016). Chronic kidney disease in low- and middle-income countries. Nephrol Dial Transplant.

[17] Pereira BJ (2000). Introduction: new perspectives in chronic renal insufficiency. Am J Kidney Dis.

[18] Orlandi PF, Huang J, Fukagawa M, Hoy W, Jha V, Oh KH, et al. Feldman HI; iNET-CKD collaborators. A collaborative, individual-level analysis compared longitudinal outcomes across the international network of chronic kidney disease (iNETCKD) cohorts. Kidney Int. 2019;96(5):1217–33. 10.1016/j.kint.2019.07.024. 10.1016/j.kint.2019.07.024 31570197

[19] Chang H, Hawley NL, Kalyesubula R, Siddharthan T, Checkley W, Knauf F, et al. Challenges to hypertension and diabetes management in rural Uganda: a qualitative study with patients, village health team members, and health care professionals. Int J Equity Health. 2019;18(1):38. 10.1186/s12939-019-0934-1. 10.1186/s12939-019-0934-1 PMC6394065 30819193

[20] Chamie G, Kwarisiima D, Clark TD, Kabami J, Jain V, Geng E, et al. Uptake of community-based HIV testing during a multi-disease health campaign in rural Uganda. PLoS One. 2014;9(1):e84317. 10.1371/journal.pone.0084317. 10.1371/journal.pone.0084317 PMC3879307 24392124

[21] Pastorius Benziger C, Bernabe-Ortiz A, Miranda JJ, Bukhman G (2011). Sex differences in health care-seeking behavior for acute coronary syndrome in a low income country. Peru Crit Pathw Cardiol.

[22] J Jeet G, Thakur JS, Prinja S, Singh M (2017). Community health workers for non-communicable diseases prevention and control in developing countries: Evidence and implications. PLoS One.

