# Supplementary figures and images for: The rural Uganda non-communicable disease (RUNCD) study: prevalence and risk factors of self-reported NCDs from a cross sectional survey
Source: BMC Public Health. 2021 Nov 7;21:2036. doi: 10.1186/s12889-021-12123-7 (PMC8572568; doi:10.1186/s12889-021-12123-7)

**A — Female**

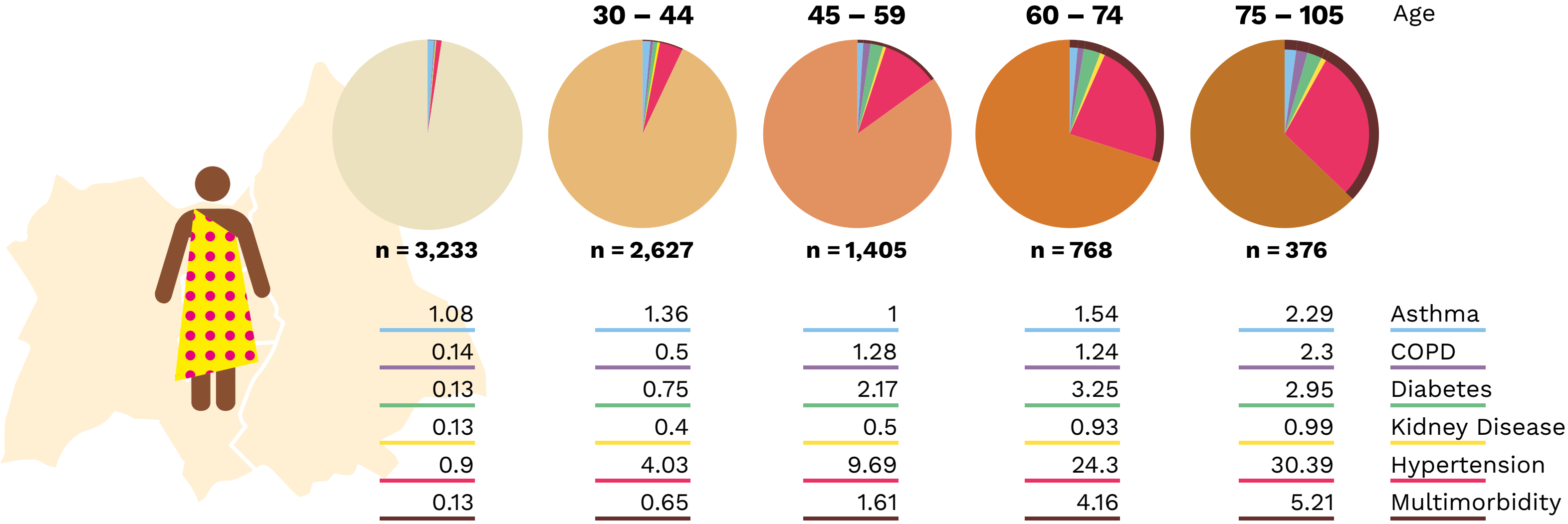

**B — Male**

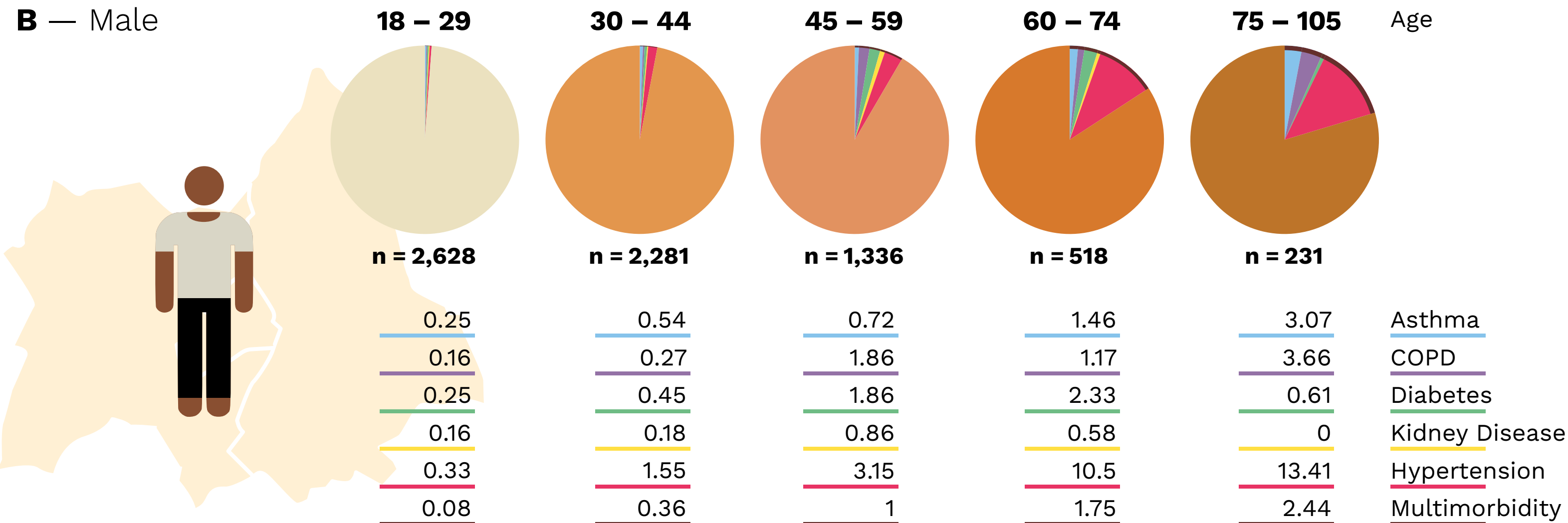

**C — Overall**

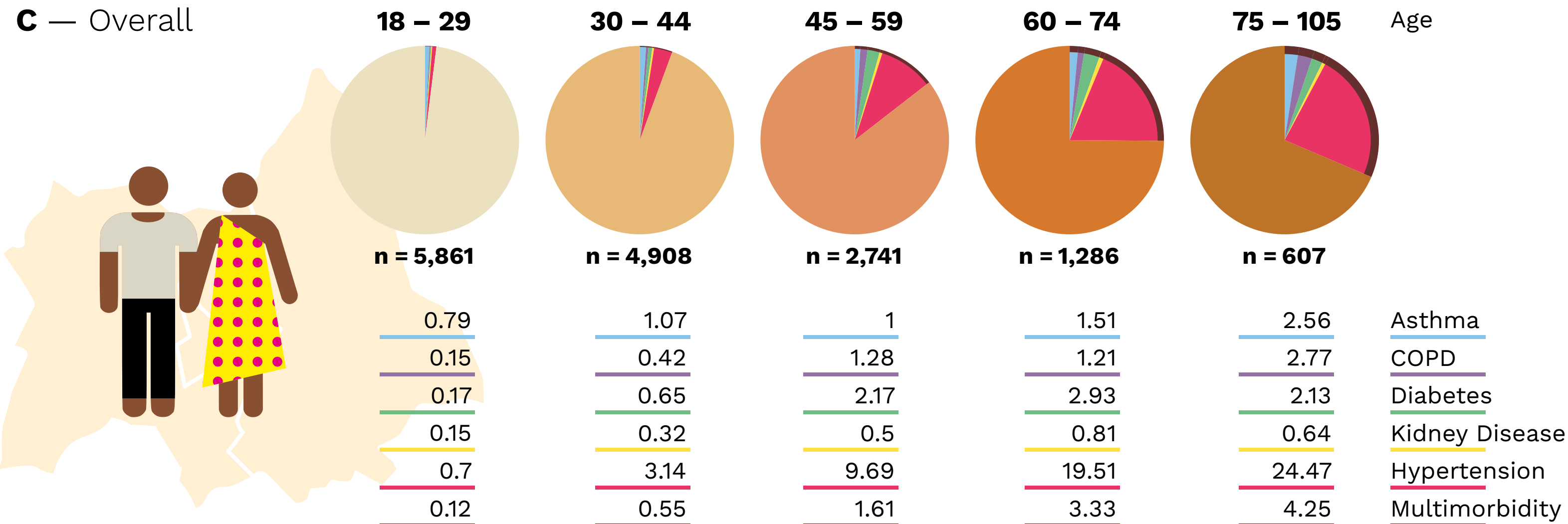

Supplement: Supplementary file 1 — Additional file 1: Supplement Fig. 1. Prevalence of self-reported non-communicable diseases in Nakaseke stratified by sex and age. The prevalence of self-reported asthma, COPD, diabetes, kidney disease, hypertension, or multimorbidity by 15-year increments stratified by sex. (Designed by Helmut Kraus, permission obtained) [file 12889_2021_12123_MOESM1_ESM.pdf]
